# Cognitive Abilities and Academic Achievement as Intercultural Competence Predictors in Russian School Students

**DOI:** 10.3390/jintelligence10020025

**Published:** 2022-04-26

**Authors:** Irina A. Novikova, Marina V. Gridunova, Alexey L. Novikov, Dmitriy A. Shlyakhta

**Affiliations:** 1 Psychology and Pedagogy Department, Peoples’ Friendship University of Russia (RUDN University), 6 Miklukho-Maklaya Str., Moscow 117198, Russia; la-destinee@yandex.ru (M.V.G.); shlyakhta-da@rudn.ru (D.A.S.); 2 General and Russian Linguistics Department, Peoples’ Friendship University of Russia (RUDN University), 6 Miklukho-Maklaya Str., Moscow 117198, Russia; novikov-al@rudn.ru

**Keywords:** intercultural competence, intercultural sensitivity, cognitive abilities, academic achievements, secondary school students, gender differences

## Abstract

The development of intercultural competence (ICC) is important for the modern personality in an unstable and diverse world, but there is a lack of research on this phenomenon in the context of age, gender and intellectual differences. The purpose of the present exploratory study is to identify relations between ICC, cognitive abilities and academic achievements among Russian school students. The sample included 106 (55% female) students in the 9th grade of Moscow secondary school. ICC was measured with the author’s modification of *The Intercultural Sensitivity Scale* by Khuhlaev and Chibisova, developed on the basis of the Developmental Model of Intercultural Sensitivity by Bennett. Cognitive abilities were determined with the *School Test of Intellectual Development* by Akimova et al. Academic achievements were evaluated using GPA. The findings of our research show that: (1) higher academic achievements and cognitive abilities usually characterize schoolchildren, who are not inclined to absolutize cultural differences and do not consider them to be barriers to intercultural interaction; (2) the most significant predictors of ICC features from the studied cognitive abilities are *analogy* and *generalization*, but *generalization* has opposite impacts in male and female students. This fact should be taken into account in the context of ICC developments, especially in male school students prone to ethnocentrism.

## 1. Introduction

In the last 2–3 years, humanity has faced a number of rapid, significant and irreversible changes that are currently taking place around the world. The COVID-19 pandemic has forced almost every person to adapt to unknown conditions in a situation of high uncertainty and instability (Koffman et al. 2020; Kondratyuk et al. 2021; etc.). In this light, the issue of intercultural interaction not only has not lost its relevance but, on the contrary, has acquired new perspectives. On the one hand, the introduction of strict restrictions at the beginning of the pandemic led to a sharp decrease in the number of face-to-face intercultural interactions. However, on the other hand, the need to maintain communication during the lockdown has led to the explosion of online technologies, which has erased physical boundaries in communication between countries and cultures: it has suddenly become commonplace to “meet” a representative of another country at a business meeting or even at home. At the same time, the availability of online interaction does not eliminate the influence of the intercultural context, and it is still required to take into account the cultural characteristics of the interlocutor to show appropriate intercultural qualities and skills.

Thus, the development of *intercultural competence* (ICC) is important both for improving the efficiency of work processes and for obtaining education and expanding social ties, that is, for solving important problems not only for adults but also for children, starting from school age. The growth of globalization and migration processes in the world means that children of different nationalities and ethnic groups are studying together in the school classroom more and more often (Darder 2012; Faas 2011; Kytina and Khamraeva 2021). According to the UN, “the number of persons living outside their country of birth or citizenship reached 281 million in 2020, up from 173 million in 2000 and 221 million in 2010” (United Nations 2020). In 2020, the Russian Federation ranked fourth in the world in terms of the number of migrants—12 million (United Nations 2020). Quite recently, the results of sociological research showed that in Moscow and the Moscow region, besides Russians, representatives of 56 ethnic groups, including 7–16% of migrants, were studying in schools (Alexandrov et al. 2015), and it can be assumed that in the future, this number will increase.

In turn, the basis for the development of ICC is its theoretical models, as well as the results of empirical studies of its factors and predictors. In Western science, the problem of ICC and similar concepts (such as cultural competence, intercultural communicative competence, intercultural sensitivity, cultural intelligence, multicultural effectiveness, etc.) began to be studied after the Second World War; in Russian psychology, it began only at the end of the 20th century (Stewart 1977; Bartel-Radic 2009; Varhegyi and Nann 2011; Sadokhin 2016; Chernyak 2015; Gridunova 2018). Since then, dozens of ICC models have been developed by psychologists, educators, sociologists, linguists and specialists from related disciplines from different countries within the framework of different approaches and models (Leung et al. 2014; Chernyak 2015; Gritsenko et al. 2020a, 2020b; Khukhlaev et al. 2021). Due to the fact that there is no generally accepted definition of ICC or a unified approach to its study, we, following Whaley and Davis (2007), propose to consider this concept in the broadest sense, as a generalized definition of the phenomenon characterizing personality activity and efficiency in a multicultural environment in terms of the different aspects of intercultural diversity and dialogue.

In modern publications on the ICC phenomenon, its models and diagnostic and development tools, as well as research on various factors and predictors of ICC, are discussed (Barrett 2018; Bartel-Radic and Giannelloni 2017; Borghetti 2017; Leung et al. 2014; Wagner et al. 2017; Gritsenko et al. 2021; Khukhlaev et al. 2021; etc.). The most popular ICC models developed in Western psychology and used for research around the world include the Development Model of Intercultural Sensitivity (DMIS) by Bennett (Bennett 1993; Hammer et al. 2003); the Intercultural Communicative Competence Model by Byram (1997); the Cultural Intelligence Model by Earley and Ang (Ang et al. 2006); etc. These models are well known in Russian psychology, and recently, based on these and other Western models of ICC (Gritsenko et al. 2020a, 2020b), as well as on the anxiety/uncertainty management theory of Gudykunst (Gudykunst 2005; Gudykunst and Nishida 2001), the Russian psychologist Khukhlaev and colleagues developed and tested the Comprehensive Model of Intercultural Competence (Khukhlaev et al. 2020).

To the best of our knowledge, the socio-cultural and socio-psychological factors of ICC have been studied quite extensively in different samples, while individual and personality factors have been studied less frequently, especially in Russian psychology.

Thus, gender and age, which are regarded as two of the main individual factors of intercultural interaction and adaptation (Stefanenko 1994), are less represented in ICC studies. For example, Hammer, Bennett and Wiseman, the creators of the famous Intercultural Development Inventory (IDI) developed on the basis of the DMIS by Bennett, noted that they did not reveal the influence of gender differences (Hammer et al. 2003). Subsequently, conflicting results were obtained: on the one hand, Kehl and Morris (2008), based on a meta-analysis, concluded that female students are more susceptible to the influence of studying abroad compared with boys, as their thinking becomes more global, but on the other hand, according to Wang and Ching (2015), some indicators of intercultural effectiveness, in particular, “relaxation in interaction” and “management of interaction”, are higher among young men. Further research on intercultural competence, intercultural sensitivity and intercultural effectiveness also showed contradictory results. Morales (2017) did not confirm that gender does not significantly impact intercultural sensitivity; Demircioğlu and Çakir (2016) maintained that gender is a relevant variable with a significant impact on intercultural sensitivity, and Tompkins et al. (2017) revealed that women’s intercultural competence is significantly higher than men’s intercultural competence. Bećirović et al. (2019), using *The Intercultural Effectiveness Scale* (IES), showed that on two subscales, the results were significantly higher in female participants, and on one subscale, the results were higher in male participants; on the other three subscales and for the IES overall score, there were no significant gender differences. 

In Russian ICC studies based on the modified version of DMIS, it was shown that male university students are more inclined to absolutize intercultural differences, i.e., ethnocentrism (Logashenko 2015; Gridunova 2018), but, in general, gender differences are not very significant (Novikova et al. 2020). In other Russian studies based on the model by Barrett (2018), it was revealed that “for women, the success of intercultural interaction depends on practical skills, self-education, the ability to listen and understand, readiness for cooperation, tolerance; and for men—from respect and responsibility, adaptability, knowledge and their critical reflection” (Makhmutova et al. 2020).

Due to the fact that most of the ICC studies of intercultural competence were carried out on samples of university students and older individuals, the age characteristics of ICC have not been studied much yet (Logashenko 2015; Wagner et al. 2017; Gridunova 2018). According to Manoilova (2011), the multi-ethnic competence of adults (teachers) in comparison with university students can be characterized as higher and having a more harmonious structure. The study by Gridunova (2018) did not reveal significant differences in the indicators of intercultural sensitivity between school and university students, although university students have a more consistent structure of ICC.

Personality traits from the Five-Factor Model (FFM) or the “Big Five” (neuroticism, extraversion, openness to experience, agreeableness and conscientiousness) are often considered as factors and predictors of ICC and similar concepts in international (Hofstede and McCrae 2004) and, more recently, Russian psychology (Gridunova et al. 2017; Gridunova 2018; Novikova et al. 2017; Novikova et al. 2019; Novikova et al. 2020). Numerous international studies have shown that openness to experience (the FFM personality trait that is often associated with intelligence and creativity) is the most universal predictor of ICC and similar concepts compared to other traits (Bakalis and Joiner 2004; Huang et al. 2005; Wang and Ching 2015; etc.). It is important that relationships between cultural intelligence and openness are revealed: for example, among all of the FFM personality traits, only openness correlates with each of the four aspects of cultural intelligence (Ang et al. 2006), and openness in combination with some components of cultural intelligence increases efficiency in an intercultural context (Soldatova and Chigarkova 2016). In previous research, the authors found that the majority of FFM personality traits correlate with ICC indicators in Russian university and school students (Gridunova et al. 2017; Gridunova 2018; Novikova et al. 2017; Novikova et al. 2019). In particular, the FFM personality traits more strongly affect ICC features in school students than in university students, and a relatively universal predictor of ICC features in both groups of Russian students is conscientiousness (Novikova et al. 2019).

Van der Zee and Van Oudenhoven (2014) also described two groups of personality traits (not from the FFM) that are associated with multicultural success or multicultural effectiveness:

(1) Traits associated with stress (emotional stability and flexibility): they correlate with a lower tendency to perceive intercultural situations as threatening, act as a defense mechanism against cultural shock and prevent the rigidity of cultural identity;
(2) Social-perceptual traits (social initiative, cultural empathy and openness): they are associated with the perception of intercultural situations as a challenge, promote cultural learning and facilitate identification with a new culture (Van der Zee and Van Oudenhoven 2014).

Among the personality factors of intercultural interaction, abilities have also been studied, which is especially characteristic of the competence-based approach: ICC is considered one of the abilities of the personality (Apalkov 2011; Ptitsyna 2008; Varhegyi and Nann 2011). Knapp (1995) identified three types of abilities that determine competence in intercultural communication: (1) the ability to manage psychological stress (management of frustration, fears and loneliness; tolerance for uncertainty), (2) the ability to build interpersonal relationships (empathy, lack of prejudice, and the ability to enter into contact) and (3) communication skills.

Ting-Toomey (1999) identified seven attributes of ICC and the corresponding abilities: tolerance for uncertainty—the ability to consciously respond to new situations; openness of consciousness—the ability to non-judgmentally relate to the culture of others; cognitive flexibility—the ability to “shift the starting point”; respectful attitude—the ability to show a respectful and positive attitude towards another; situational adaptability—the ability to effectively adapt to a specific situation; verbal and non-verbal sensitivity—the ability to express empathy verbally and non-verbally; and creative thinking—the ability to think divergently and systematically.

Many authors, in the context of research on intercultural competence, talk about the ability to communicate, resolve conflicts and show empathy; awareness of ethnic stereotypes; and the identification of intercultural similarities and differences, including non-verbal (Bush et al. 2001; Apalkov 2011; Deardorff 2006; Ting-Toomey 1999; Varhegyi and Nann 2011; Gudykunst 2005; Ptitsyna 2008; Logashenko 2015).

However, we know of much less research on the relationship between ICC and cognitive abilities, understood as the ability of the human to process, store and extract information, including processes such as attention, memory and thinking. In this connection, we can list the use of cognitive approaches in linguistics in the analysis of intercultural communication (Nurmoldayev et al. 2020), in pedagogy in consideration of culturally diverse instructional strategies (Milhouse 1995), and in international management in the study of cross-cultural training effectiveness (Kempf and Holtbrügge 2020). In psychological science, we can cite the general analysis of the cognitive aspects of intercultural competence (Lonner and Hayes 2004), as well as more specific studies on metacognition’s role in cultural learning (Morris et al. 2019); on the ability of cognitive schemas of culturally based skills in an acculturation context among Latinx adults (Driscoll and Torres 2020); and on the relationship between intercultural experience and creativity (Dunne 2017; De Prada et al. 2020). A similar study by Russian scientists Bultseva and Lebedeva (2018) revealed the links between ICC and creativity in a sample of Russian students. The results of this study showed that creativity indicators (fluency, flexibility and originality) were associated with positive attitudes towards representatives of other cultures, but they were not associated with knowledge (general knowledge about other cultures) and awareness (awareness of one’s own experience of intercultural interaction). It was also found that creativity was higher in students who had undergone cultural training, as well as in students who had experience staying abroad. These authors further revealed that intercultural competence mediated the relationship between the intercultural experiences and creativity of students (Bultseva and Lebedeva 2021). Gridunova et al. (2017) showed that ICC indicators studied on the basis of the modified version of DMIS had close correlations with intellectual abilities in Russian school students; moreover, ethnorelativistic scales had positive relationships with intellectual abilities, and ethnocentric scales had negative ones, which indicates the important role of high intellectual development in ICC development.

It is common knowledge that cognitive abilities are one of the most studied and most stable predictors of academic performance (Stadler et al. 2016; Timofeeva et al. 2015; Vilia et al. 2017; Shi and Qu 2021), although the relationships among these variables are very complex and can be mediated by many other factors (Bergold and Steinmayr 2018; Shi and Qu 2021). At the same time, correlations between cognitive abilities, academic performance and intercultural competence (or related constructs) are practically not studied. Lyubovnikova et al. (2015) demonstrated that task reflexivity was indirectly related to academic performance through intercultural sensitivity in MBA students in UK Business School; Bećirović et al. (2019) showed that GPA had a significant impact on intercultural effectiveness, as it increased with the increase in Bosnian university students’ GPAs.

Thus, based on a review of the literature, it can be argued that an important direction in the study of personality factors of ICC may be to identify its relationship with cognitive abilities and academic achievements while taking into account the gender of the participants. As we noted above, such studies will be especially valuable for schoolchildren, since, despite the practical relevance, these samples have been very poorly studied in this direction.

The purpose of the present study is to identify relations between ICC indicators based on the modified DMIS, cognitive abilities and academic achievements among Russian secondary school students. This study is exploratory and has the following research objectives:

(1) Compare indicators of ICC, cognitive ability and GPA between male and female school students;
(2) Consider the correlations between ICC indicators, cognitive abilities and academic achievements in Russian male and female school students;
(3) Consider the cognitive abilities and academic achievements as ICC predictors and compare their impacts on male and female Russian secondary school students.

Based on previous studies of students’ ICC, we assume that there are gender differences both in the manifestations of ICC indicators and in the associations of these indicators with cognitive abilities and academic achievements among school students.

## 2. Materials and Methods

### 2.1. Participants

A total of 121 (55% female) students in 9th grade of Moscow secondary school, aged 15 to 16 (the mean is 15.60 ± .47 years), took part in the research. They participated in the study during classes with a school psychologist. They were advised that participation would be free and voluntary. In the final sample, we included 106 participants (51 boys and 55 girls) who correctly completed all techniques used.

The study was conducted in accordance with the APA Ethical Standards and the Code of Ethics of the RPS (Russian Psychological Society), and the protocol was approved by the Ethics Committee of RUDN University (#050422-0-037).

### 2.2. Techniques

In accordance with the purpose of the study and research hypotheses, we used three diagnostic tools.

To diagnose ICC features, *The Intercultural Sensitivity Scale* (ISS) by Chibisova and Khukhlaev (2008) in Y.A. Logashenko’s modified version (Logashenko 2015) was used. The original version of this scale was developed based on the DMIS by Bennett in the Russian modification (Chibisova and Khukhlaev 2008). It contains 51 items, which are grouped into four subscales corresponding to different “orientations toward cultural difference”: from ethnocentrism to ethnorelativism (Bennett 1993). *Minimization* and *Absolutization* correspond to ethnocentric orientations according to the DMIS, *Ambivalence* is a transitional orientation from ethnocentrism to ethnorelativism, and *Acceptance* corresponds to ethnorelative orientations according to the DMIS (Bennett 1993; Hammer et al. 2003). We reduced the number of items in each subscale to 8 based on a psychometric study using the coefficients Cronbach’s α and McDonald’s ω. All subscales of the ISS modified by us have acceptable Cronbach’s α coefficients (.60–.78) and McDonald’s ω_h_ coefficients (.47–.76), which indicates their acceptable internal consistency (Zinbarg and Revelle 2009). In the final version of the ISS, the total raw scores for each subscale can range from 0 to 80 points. This version of the questionnaire has been well tested on different samples of Russian university and school students in our previous research (Gridunova et al. 2017; Gridunova 2018; Novikova et al. 2017; Novikova et al. 2019; Novikova et al. 2020).

To determine cognitive abilities, the normative *School Test of Intellectual Development* (STID-2) by M.K. Akimova et al. was used (Akimova 2005). STID-2 is one of the most used tools for diagnosing cognitive abilities in school students at Russian secondary schools. STID-2 includes the following subtests:

(1) *Scientific and cultural awareness* (20 tasks to choose one of five options for correct sentence completion);
(2) *Public and political awareness* (20 tasks to choose one of four synonyms for a given word);
(3) *Analogy* (25 tasks to choose one of five concepts that has the same logical connection with the given word as a pair of initial concepts);
(4) *Classification* (20 tasks to exclude one of five proposed concepts that is not related to the rest of the common features);
(5) *Generalization* (19 tasks to find a common feature between two concepts and write it down);
(6) *Spatial representations* (5 tasks choose the set from four options whose parts make up the given figure and 5 tasks to choose one of four three-dimensional figures whose image sweep is given in the condition of the task).

The maximum time to complete all subtest tasks is 54 min. The sum of scores for each subtest gives the *total score* for the STID-2 and is a criterion for determining the level of development of cognitive abilities. There are 4 levels for students in grade 9: (1) below the age norm (0–45 points), (2) age norm (46–70 points), (3) high age norm (71–100 points) and (4) above the age norm (101–148 points). The STID-2 has two parallel forms, A and B, and has the following psychometric characteristics: the equivalence of parallel forms for the total score is .83; homogeneity is .96–.98; and retest reliability for the *total score* is .93 for form A and .90 for form B. Correlation coefficients with the results of the Intelligence Structure Test (IST) by Amthauer are .75; correlation coefficients with school performance are .57 (form A) and .54 (form B) (Akimova 2005, p. 129).

To assess academic achievements, we used grade point averages (GPAs) for all subjects in the 9th grade provided by the school.

### 2.3. Statistical Analysis

The descriptive statistics methods, coefficients Cronbach’s *α* and McDonald’s *ω*, Spearman’s rank correlation analysis, Wilcoxon rank-sum test with continuity correction, Fisher *F*-test and multiple regression analysis were used for statistical analysis. Most of the studied variables have a normal distribution according to the Anderson–Darling criterion, but two STID subtests (*scientific and cultural awareness* and *public and political awareness*) have a negative right-sided skewness (respectively −.71 and −.96, *p* < .001). In this regard, non-parametric methods were chosen for correlation and comparative analyses that correspond to this type of data.

Regression analysis was performed by using the method of “backward” stepwise search. Independent variables were cognitive abilities (six STID-2 subtests) and GPA; dependent variables were ICC features (four ISS subscales). In the first step, full regression models with all possible predictors of each ISS subscale were constructed for the general sample and separately for male and female students. The next step involved analyzing all of the input models by searching all possible predictor combinations and evaluating the informational contribution of each set using the Akaike An Information Criterion (AIC). Models having the highest information load for the smallest number of predictors (“a best predictor model”) were selected for further analysis. Statistical processing was carried out in the R software environment for statistical computing and graphics, version 4.1.1 (R Core Team 2021; Revelle 2019; The Jamovi Project 2021; Epskamp et al. 2012).

## 3. Results

Table 1 presents the results of descriptive statistics (means and standard deviations) and the analysis of differences between all variables studied using the *W-*test in male and female school students.

Table 1 shows that there are significant differences for most ISS scales: *Acceptance* (ICC ethnorelativistic subscale) and *Minimization* (ICC ethnocentric subscale) are higher in female students, but *Absolutization* (ICC ethnocentric subscale) is higher in male students. Among cognitive abilities, only *spatial representations* are slightly higher in female students. GPA is significantly higher for female school students.

**Figure 1:**
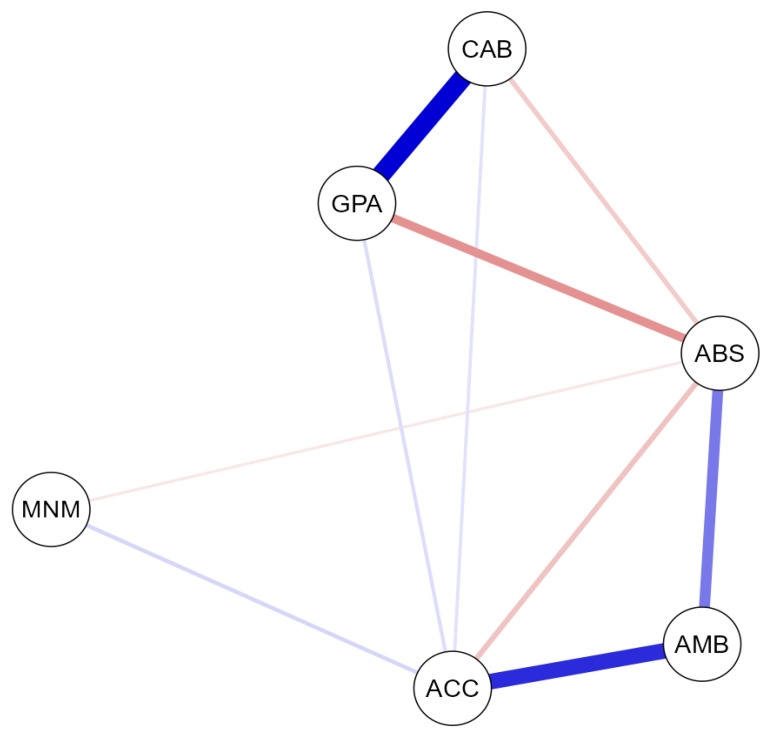
Graphical representation of the correlations between ISS subscales, STID-2 total score and GPA in general sample of school students (*N* = 106). *Note*: Blue lines are positive correlations, and red lines are negative correlations; line thickness corresponds to the value of the correlation coefficient. ACC is *Acceptance*, AMB is *Ambivalence,* ABS is *Absolutization,* and MNM is *Minimization*; CAB is cognitive abilities (total score of STID-2).

**Figure 2:**
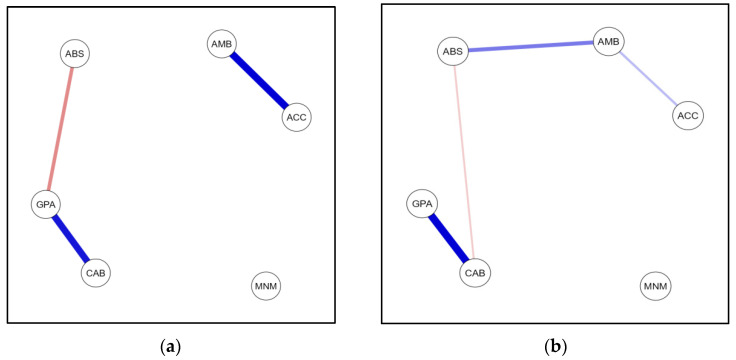
Graphical representation of the correlations between ISS subscales, STID-2 total score and GPA in male (**a**) and female (**b**) school students. *Note*: Blue lines are positive correlations, and red lines are negative correlations; line thickness corresponds to the value of the correlation coefficient. ACC is *Acceptance*, AMB is *Ambivalence*, ABS is *Absolutization*, and MNM is *Minimization*; CAB is cognitive abilities (total score of STID-2).

Table 2, Table 3 and Table 4 present Spearman’s correlations between ISS subscales, STID-2 subtests and total score, and GPA. Figure 1 and Figure 2 visualize these correlations (Epskamp et al. 2012). Due to the fact that all STID-2 subtests positively correlate with each other and with the STID-2 total score, we include only the total score as the cognitive ability indicator in the visualizations.

Table 2 and Figure 1 show that among ICC indicators, *Acceptance* (ICC ethnorelativistic subscale) has positive but non-significant correlations with GPA and cognitive abilities, while *Absolutization* (ICC ethnocentric subscale) has stronger negative correlations with GPA and cognitive abilities (STID-2 total score) in the general sample. Cognitive abilities have an expected strong positive correlation with GPA.

Table 3 and Table 4 and Figure 2 show that among ICC indicators, only *Absolutization* (ICC ethnocentric subscale) has a trend towards a negative correlation (*p* < .10) with GPA in male students and a negative correlation with cognitive abilities (STID-2 total score) in female students. Cognitive abilities have an expected strong positive correlation with GPA in both groups.

The results of the multiple regression analysis (best predictor models) are presented in Table 5, Table 6 and Table 7. Multiple correlation coefficients between dependent variables (ICC features, i.e., ISS subscales, except for *Minimization*) and predictors (six STID-2 subtests and GPA) for most of the models are statistically valid according to the Fisher *F*-test, which confirms that there is a significant impact of some cognitive abilities and/or GPA on ICC indicators. At the same time, there is a large range of the adjusted determination coefficients (R^2^_adj_), which reflects the different degrees of these predictors’ impacts on different ISS subscales.

Table 5 shows that the best predictor model for *Acceptance* predicts 6.6% of the variance in the general sample, 16.4% in male students and only 2.9% in female students. The only significant predictor of *Acceptance* (ethnorelativistic subscale) in the general sample is *analogy*. A paradoxical result was obtained in the male student sample: *scientific and cultural awareness* has a significant negative impact on *Acceptance*, but in the female sample, this impact has the opposite sign (but this model does not reach the level of significance, *p* = .11).

Table 6 shows that regression models for *Ambivalence* (transition stage from ethnocentrism to ethnorelativism) predict only 2.9% of the variance in the general sample, 13.1% in male students and 5.3% in female students. GPA has a significant negative impact on *Ambivalence* in general and male samples. *Generalization* has the opposite impact on *Ambivalence* in male (positive) and female (negative) samples.

Table 7 shows that the regression models with the highest adjusted determination coefficients (R^2^_adj_ = 15.2–17.4%) were obtained for *Absolutization* (ethnocentric subscale) in all studied samples. Only GPA has a strong negative impact on *Absolutization* in the general sample and in the male sample. *Generalization* has a negative impact on *Absolutization* in the female sample, as in the case of *Ambivalence*.

## 4. Discussion

The purpose of this exploratory study is to identify relations between ICC indicators based on the modified DMIS, cognitive abilities and academic achievements among Russian secondary school students while taking into account the gender of the participants. Based on the results of studies in which it was shown that such personality features as traits (openness, flexibility, cultural empathy, etc.) and different abilities are associated with ICC and can be its predictors, and based on our previous ICC studies, we expected that there would be correlations between ICC, cognitive abilities and academic achievements and that they may differ between male and female school students. Summarizing the results of the study, we can conclude that our assumptions are partly confirmed.

Firstly, we showed that there are significant gender differences in ICC: male students tend to overestimate and absolutize cultural differences, while female students tend to both underestimate cultural differences and take them into account in intercultural interactions. These data support the view of researchers who have previously shown significant gender differences in intercultural competence, intercultural sensitivity, intercultural effectiveness and similar phenomena (Bećirović et al. 2019; Demircioğlu and Çakir 2016; Gridunova 2018; Kehl and Morris 2008; Logashenko 2015; Makhmutova et al. 2020; Tompkins et al. 2017; Wang and Ching 2015). Most of all, our data are consistent with conclusions about the contradictory nature of these differences reported by Bećirović et al. (2019): we found that the ISS ethnocentric scale *Absolutization* is higher in males, while both ethnorelativistic (*Acceptance*) and ethnocentric (*Minimization*) scales are higher in females, and there are no gender differences on the “transitional” scale *Ambivalence*. When evaluating these results, it should be taken into account that previous studies involved university students and older participants, not school students, as in our case. As for cognitive abilities, we did not reveal gender differences in most subscales or in the total score of STID-2, which corresponds to the data of the test developers, who did not provide information about such differences (Akimova 2005). We also showed that GPA is significantly higher among female students, which is consistent with common belief and the results of some studies (Timofeeva et al. 2015), although there are no official open statistics on this issue in Russia.

Secondly, we found significant negative correlations only for *Absolutization* (ICC ethnocentric subscale) with both cognitive abilities (STID-2 total score) and GPA, and only in the general sample: there are no clear gender differences in the correlations of the studied variables, which we assumed based on our previous studies of university students. According to these data, higher academic achievements and cognitive abilities usually characterize both male and female schoolchildren, who are not inclined to absolutize cultural differences and do not consider them to be insurmountable barriers to intercultural interaction. *Acceptance* (ISS ethnorelativistic subscale), on the contrary, tends to have positive correlations with GPA and cognitive abilities (STID-2 total score), but with less significance. Consequently, school students who tend to take into account and accept cultural differences in the process of interaction, as a rule, have better academic performance and cognitive abilities. In general, these results are in line with data on the positive role of cognitions and various cognitive abilities (e.g., creativity) in the development of intercultural competence and related constructs (Bultseva and Lebedeva 2018; De Prada et al. 2020; Driscoll and Torres 2020; Dunne 2017; Morris et al. 2019).

Finally, we revealed that cognitive abilities and/or academic achievements could explain from 2.9 to 17.4% of the ICC indicators’ variance on the basis of the DMIS (*Acceptance*, *Ambivalence* and *Absolutization*). Such determination coefficients are not considered to be very high, yet the result can be deemed satisfactory, given the huge number of external and internal factors influencing ICC indicators. In addition, it was shown that the most significant predictors of ICC features from the studied cognitive abilities are *analogy* and *generalization*, but *generalization* has opposite impacts in male and female students. We can speculate that *analogy*, as the cognitive ability to transfer similar meaning from one object or situation to others (Akimova 2005; Rogov 1999, p. 162), helps to see similarities even across cultures and thus promotes acceptance of cultural differences. In this regard, the development of the ability to analogize can probably contribute to the development of ethnorelativism.

Generalization, as the cognitive ability for abstraction, is considered “the process of deriving a concept, judgment, principle, or theory from a limited number of specific cases and applying it more widely, often to an entire class of objects, events, or people” (APA 2022). The *generalization* subtest of STID-2 includes open-ended questions, the answers to which are evaluated according to the quality of generalizations: 2 points for the correct answers given in the table, 1 point for both a broader categorical generalization and a narrower particular generalization, and 0 points for an incorrect answer (Akimova 2005; Rogov 1999, p. 161). In this regard, we are inclined to explain the opposite impact of *generalization* on the *Ambivalence* subscale (transition stage from ethnocentrism to ethnorelativism) by broader categorical generalizations in female students, which, in turn, contribute to their more coherent and harmonious orientations in relation to cultural differences. This assumption is confirmed by the findings by Katvalyan and Kostromina (2018) that Russian girls aged 12 to 15, to a lesser extent than boys, distinguish ethnic features in their self-concepts.

The paradoxical results showing the opposite impact of *scientific and cultural awareness* on the *Acceptance* of cultural differences in female and male samples, in our opinion, can be explained in a similar way. Moreover, studies are known (for example, Sapolsky 2017) that describe the significant role of physiological processes in the manifestation of nationalistic sentiments and in shaping the perception of people around us through the prism of the “we”–“them” dichotomy. Perhaps male adolescents, who are more scientific and culturally aware and, therefore, better able to recognize signs of other cultures, are more likely to direct their pubertal aggression towards people whom they perceive as “them”.

In addition, we revealed that GPA, as an indicator of academic achievements, which may depend on both cognitive and social factors, can explain 13.1–16.9% of the variance in the ICC ethnocentric subscale (*Absolutization*) in the general and male school student samples as the only “best predictor.” Together with cognitive abilities, GPA also has a significant negative association with the *Ambivalence* subscale (transition stage from ethnocentrism to ethnorelativism) in the male and general samples. Accordingly, school students (especially males) with lower academic achievements tend to exaggerate cultural differences and perceive them as barriers to intercultural interaction. These data are consistent with the findings reported by Lyubovnikova et al. (2015) that task reflexivity was related to academic performance through intercultural sensitivity in MBA students in UK Business School, and the results reported by Bećirović et al. (2019) that GPA had a significant positive impact on intercultural effectiveness in Bosnian university students.

Thus, with the help of regression analysis, we largely confirmed the conclusions of previous studies on the positive role of various cognitive abilities in the development of intercultural competence and related phenomena (Bultseva and Lebedeva 2018; De Prada et al. 2020; Driscoll and Torres 2020; Dunne 2017; Morris et al. 2019); however, we identified gender differences that were previously poorly studied in this context. The specificity of our study is also that the sample includes schoolchildren, but not university students, who are studied much more often.

Thus, we have shown that there are gender differences both in the manifestations of ICC indicators among school students and in the associations of these indicators with cognitive abilities and academic achievements. The findings of our study should be used in psychological support programs for schoolchildren studying in a multicultural environment to develop their ICC. In this direction, it will be useful to develop and improve the cognitive abilities of school students and pay special attention to male students with low academic performance.

There are several limitations to our study that should be taken into account when evaluating its results and conducting future research in this area. Firstly, the main limitation is the relatively small sample size, which, perhaps, did not allow us to obtain more significant results. Another possible limitation of this study is the measure used to collect the data. The ICC can be measured with various tools, the most popular being self-questionnaires. However, there is a need to use more objective methods, including expert assessments and quasi-experiments. The next limitation is a certain lack of prior research studies on ICC predictors in school students both in international and Russian psychology, so it is difficult to compare our results with other researchers’ and provide a more comprehensive outlook on the problem.

Summing up all of the findings and limitations of our research, we can determine its future prospects: (1) the complex study of different ICC predictors (social attitudes, personality traits, intelligence level, cognitive abilities, creativity, etc.); (2) a sample expansion and the study of not only schoolchildren but also university students; (3) the use of additional measurement methods for ICC, as well as other methods of statistical analysis; and (4) the development of programs to improve ICC in school students in a multicultural educational environment, taking into account their cognitive abilities and academic achievements.

## Figures and Tables

**Table 1:** Means (M), standard deviations (SD) and Wilcoxon’s rank-sum test with continuity correction (*W*-test) between study variables in male and female school students.

Variables	General Sample (*N* = 106)		Male (*N* = 51)		Female (*N* = 55)		Wilcoxon’ *W*-Test	*p*-Level
	M	SD	M	SD	M	SD		
Orientations toward Cultural Difference (ISS subscales)								
Acceptance	53.21	13.97	47.88	15.10	58.15	10.82	1951.5	.000 ***
Ambivalence	51.18	12.41	50.31	12.29	51.98	12.59	1542	.379
Absolutization	36.17	15.00	40.49	14.65	32.16	14.32	921.5	.002 **
Minimization	52.37	10.59	49.61	9.37	54.93	11.09	1821	.008 **
Cognitive Abilities (STID-2 subtests)								
Scientific and cultural awareness	13.83	3.62	13.31	3.67	14.31	3.53	1629.5	.150
Public and political awareness	14.52	3.94	13.82	4.34	15.16	3.44	1638	.135
Analogy	13.53	5.24	12.80	5.67	14.20	4.77	1586	.246
Classification	12.05	3.27	11.61	3.49	12.45	3.04	1562	.313
Generalization	11.94	6.07	11.37	5.63	12.47	6.45	1525.5	.438
Spatial representations	4.87	2.22	4.43	2.30	5.27	2.09	1718.5	.044 *
Total score	77.71	22.26	73.37	22.82	81.73	21.15	1697	.063
Academic Achievement								
GPA	4.06	.52	3.84	.40	4.27	.53	2072	.000 ***

* *p* < .05; ** *p* < .01; *** *p* ≤ .001.

**Table 2:** Spearman’s correlations between studied variables in general sample of school students (*N* = 106).

Variables	1	2	3	4	5	6	7	8	9	10	11	12
1. Acceptance	—											
2. Ambivalence	.53 ***	—										
3. Absolutization	−.16	.28 *	—									
4. Minimization	.16	.05	−.14	—								
5. Scientific and cultural awareness	.12	−.06	−.27	.04	—							
6. Public and political awareness	.16	.02	−.24	.01	.77 **	—						
7. Analogy	.24	.05	−.32 *	.05	.71 **	.72 **	—					
8. Classification	.22	.02	−.23	.03	.61 **	.60 **	.64 **	—				
9. Generalization	.14	−.04	−.27	−.07	.68 **	.61 **	.66 **	.56 **	—			
10. Spatial representations	.13	.08	−.16	.06	.44 **	.36 *	.43 **	.38 **	.37 **	—		
11. Total score of STID-2	.21	−.01	−.31 *	.02	.88 **	.82 **	.84 **	.75 **	.80 **	.54 **	—	
12. GPA	.21	−.16	−.43 **	.11	.59 **	.49 **	.62 **	.44 **	.52 **	.34 *	.64 **	—

* *p* ≤ .05; ** *p* ≤ .01; *** *p* ≤ .001, corrected with Holm correction method.

**Table 3:** Spearman’s correlations between studied variables in the sample of male school students (*N* = 51).

Variables	1	2	3	4	5	6	7	8	9	10	11	12
1. Acceptance	—											
2. Ambivalence	.63 **	—										
3. Absolutization	−.09	.17	—									
4. Minimization	−.03	−.09	−.21	—								
5. Scientific and cultural awareness	.04	.07	−.16	.07	—							
6. Public and political awareness	.15	.18	−.15	.03	.78 **	—						
7. Analogy	.30	.20	−.31	.16	.62 **	.73 **	—					
8. Classification	.29	.20	−.26	−.01	.59 **	.70 **	.60 **	—				
9. Generalization	.24	.20	−.15	−.08	.69 **	.64 **	.64 **	.48 *	—			
10. Spatial representations	.09	.15	−.10	.02	.38	.40	.41	.34	.32	—		
11. Total score of STID-2	.18	.18	−.26	.03	.87 **	.89 **	.81 **	.72 **	.76 **	.46 *	—	
12. GPA	.01	−.20	−.39	.08	.52 **	.45 *	.50 *	.31	.54 **	.26	.56 **	—

* *p* ≤ .05; ** *p* ≤ .01, corrected with Holm correction method.

**Table 4:** Spearman’s correlations between all studied variables in sample of female students (*N* = 55).

Variables	1	2	3	4	5	6	7	8	9	10	11	12
1. Acceptance	—											
2. Ambivalence	.40	—										
3. Absolutization	−.01	.45 *	—									
4. Minimization	.22	.16	−.01	—								
5. Scientific and cultural awareness	.13	−.23	−.33	−.06	—							
6. Public and political awareness	.06	−.17	−.26	−.08	.75 **	—						
7. Analogy	.14	−.14	−.29	−.01	.78 **	.68 **	—					
8. Classification	.07	−.20	−.15	.01	.64 **	.49 *	.68 **	—				
9. Generalization	−.01	−.29	−.39	−.10	.67 **	.58 **	.69 **	.64 **	—			
10. Spatial representations	.08	−.01	−.11	.03	.50 *	.28	.46 *	.41 **	.41 **	—		
11. Total score of STID-2	.14	−.24	−.31	−.08	.88 **	.75 **	.85 **	.80 **	.85 **	.59 **	—	
12. GPA	.15	−.26	−.30	−.02	.63 **	.55 **	.75 **	.57 **	.58 **	.33	.71 **	—

* *p* ≤ .05; ** *p* ≤ .01, corrected with Holm correction method.

**Table 5:** Best predictor regression models for *Acceptance* subscale of ISS.

Sample/Variable	Summary of Model				Coefficients		
	R^2^_adj_	*F*	*p*-Value	Estimate	Std. Error	*t*-Value	*p*-Value
*General sample* (*N* = 106)	.066	8.405	.005				
(Intercept)				43.351	3.644	11.897	.000
Analogy				.729	.251	2.899	.005
*Male* (*N* = 51)	.164	3.457	.015				
(Intercept)				44.035	7.952	5.538	.000
Scientific and cultural awareness				−2.185	.811	−2.694	.001
Analogy				.737	.511	1.442	.156
Classification				1.334	.781	1.707	.095
Generalization				.705	.504	1.399	.169
*Female* (*N* = 55)	.029	2.636	.110				
(Intercept)				48.591	6.058	8.022	.000
Scientific and cultural awareness				.668	.411	1.624	.110

**Table 6:** Best predictor regression models for *Ambivalence* subscale of ISS.

Sample/Variable	Summary of Model				Coefficients		
	R^2^_adj_	*F*	*p*-Value	Estimate	Std. Error	*t*-Value	*p*-Value
*General sample* (*N* = 106)	.029	2.549	.083				
(Intercept)				69.895	10.112	6.912	.000
Analogy				.543	.292	1.857	.070
General point average				−6.414	2.963	−2.165	.030
*Male* (*N* = 51)	.131	3.52	.022				
(Intercept)				80.834	16.405	4.927	.000
Generalization				.829	.337	2.457	.018
Spatial representations				1.109	.755	1.47	.148
General point average				−11.679	4.755	−2.456	.018
*Female* (*N* = 55)	.053	4.021	.050				
(Intercept)				58.444	3.622	16.138	.000
Generalization				−.518	.258	−2.005	.050

**Table 7:** Best predictor regression models for *Absolutization* subscale of ISS.

Sample/Variable	Summary of Model				Coefficients		
	R^2^_adj_	*F*	*p*-Value	Estimate	Std. Error	*t*-Value	*p*-Value
*General sample* (*N* = 106)	.169	22.32	.000				
(Intercept)				85.698	10.568	8.109	.000
General point average				−12.193	2.581	−4.724	.000
*Male* (*N* = 51)	.152	9.978	.003				
(Intercept)				97.938	18.284	5.356	.000
General point average				−14.956	4.735	−3.159	.003
*Female* (*N* = 55)	.174	12.36	.001				
(Intercept)				44.196	3.847	11.489	.000
Generalization				−.965	.274	−3.515	.001

## Data Availability

The datasets used and analyzed during the current study are available from the corresponding author upon reasonable request.
